# Osteoporosis Self-Assessment Tool Performance in a Large Sample of Postmenopausal Women of Mendoza, Argentina

**DOI:** 10.1155/2013/150154

**Published:** 2013-03-04

**Authors:** Fernando D. Saraví

**Affiliations:** ^1^Bone Densitometry Unit, Nuclear Medicine School, Garibaldi 405, 5500 Mendoza, Argentina; ^2^Institute of Physiology, Faculty of Medical Sciences, Universidad Nacional de Cuyo, Mendoza, Argentina

## Abstract

The Osteoporosis Self-assessment Tool (OST) is a clinical instrument designed to select patients at risk of osteoporosis, who would benefit from a bone mineral density measurement. The OST only takes into account the age and weight of the subject. It was developed for Asian women and later validated for European and North American white women. The performance of the OST in a sample of 4343 women from Greater Mendoza, a large metropolitan area of Argentina, was assessed. Dual X-ray absorptiometry (DXA) scans of lumbar spine and hip were obtained. Patients were classified as either osteoporotic (*N* = 1830) or nonosteoporotic (*n* = 2513) according to their lowest T-score at any site. Osteoporotic patients had lower OST scores (*P* < 0.0001). A receiver operating characteristic (ROC) curve showed an area under the curve of 71% (*P* < 0.0001), with a sensitivity of 83.7% and a specificity of 44% for a cut-off value of 2. Positive predictive value was 52% and negative predictive value was 79%. The odds ratio for the diagnosis of osteoporosis was 4.06 (CI95 3.51 to 4.71; *P* < 0.0001). It is concluded that the OST is useful for selecting postmenopausal women for DXA testing in the studied population.

## 1. Introduction

Osteoporosis is a systemic skeletal disorder characterized by low bone strength (arising from both low bone mass and microarchitectural deterioration), which increases the risk of fractures. Osteoporosis is a major public health problem and an important contributor to the global burden of noncommunicable disease [1].

Currently the recommended method for the diagnosis of osteoporosis is bone mineral density (BMD) measurement by dual-energy X-ray absorptiometry (DXA) [2]. According to the World Health Organization criteria, osteoporosis is operationally defined “as a BMD that lies 2.5 standard deviations or more below the average value for young healthy women.” [2]. 

Since, due to cost and availability, DXA scans are not recommended for screening purposes, several tools based on known clinical risk factors have been developed to identify those patients with high risk of osteoporosis, in whom actual BMD testing would be most useful in terms of diagnosis, treatment, and followup [3, 4]. Some of these clinical tools, or aids in decision making, include many factors, making calculation of risk cumbersome [5, 6]. Arguably the simplest decision rule is the Osteoporosis Self-assessment Tool (OST) which only takes into account body weight and age, which in adult populations are, respectively, related inversely and directly to the risk of osteoporosis [7]. 

The OST was developed for predicting risk of femoral neck T-score at or below 2.5 in Asian postmenopausal women [8] and later validated for Caucasian European and US postmenopausal women [9]. In these populations, the performance of the OST was similar to those of more complex clinical risk assessment tools [3, 10–12] Although a related tool, called OsteoRisk, has been validated for Latin American postmenopausal women [13], no direct assessment of the OST has been yet performed in this region.

The current prevalence of osteoporosis and the incidence of osteoporotic fractures in Latin America are similar to those of Southern Europe [14–16], but lower than those of Northern Europe and the United States [1, 2]. However, a significant increase in the incidence of osteoporotic fractures is expected to occur in Latin America in the next few years, according to a World Health Organization report [2]. This highlights the need for improving clinical assessment and selection of women for BMD testing. 

In this report, the performance of the OST in a sample of postmenopausal women from western Argentina was assessed. 

## 2. Materials and Methods

### 2.1. Participants

The province of Mendoza in Western Argentina has a population of 1,742,000 inhabitants according to the 2010 census [17]. About 62% of the population lives in Greater Mendoza, the fourth largest metropolitan area of the country, which includes about 133,000 women aged 50 years or older. The current sample included 4343 women referred to the Bone Densitometry Unit of the Nuclear Medicine School for a first (diagnostic) DXA scan of lumbar spine and hip. Women with Paget's disease, primary hyperparathyroidism, or severe hip osteoarthritis were excluded. 

The research protocol was reviewed and approved by the Committee of Teaching and Research of the Nuclear Medicine School. The study was planned and conducted in full accordance with the current version (2008) of the Declaration of Helsinki.

### 2.2. Measurements and Procedures

The height and weight of each patient were measured while she stood without shoes, wearing light clothing. The body mass index (BMI) was calculated as her weight in kg divided by her height in m squared (kg/m^2^). 

Patients were asked about previous fragility fractures, glucocorticoid, estrogen or bisphosphonate treatment, a diagnosis of rheumatoid arthritis, a history of hip fracture or DXA diagnosis of osteoporosis in their parents, smoking status, alcohol intake and physical activity. Calcium intake, was assessed through a Spanish version of the food frequency questionnaire developed and validated by Magkos et al. [18], using Argentine food composition tables for the calcium content of each item included [19].

DXA scans of the lumbar spine (L1–L4) and one hip (usually the left) were performed using a Lunar Prodigy equipment (GE Healthcare Lunar, Madison, WI). Measurements were performed by one of two technicians, both of whom were certified by the International Society for Clinical Densitometry. Stability of the bone densitometer throughout the study (*in vitro *long-term precision) was checked through daily measurement of a spine phantom according to the manufacturer. Short-term *in vivo *precision was estimated by DXA scans repeated after repositioning the patient, with two measures at each site in 30 patients, according to the International Society for Clinical Densitometry Official Positions 2007 [20].

Phantom measurements showed stability of the DXA equipment throughout the study, with a coefficient of variation of 0.5%. The combined *in vivo *precision for both technicians was 1.5% for the lumbar spine, 1.8% for the femoral neck, and 1.4% for the total hip.

Patients were classified as normal, osteopenic, or osteoporotic according to the World Health Organization criteria [2], based on the lowest T-score at the lumbar spine, the femoral neck, or the total hip. Reference values were taken from the National Health and Nutrition Examination Survey (NHANES III), which is the recommended reference database for Argentine patients [21]. 

The OST score was calculated as 0.2 (weight in kg − age in years) and rounded up to the closest integer. For example, a 64-year-old woman weighing 50 kg has an OST score of 0.2  (50 − 64) = −2.8, which would be rounded up to −3, and a 52-year-old woman weighing 67 kg has an OST score of 0.2 (67 − 52) = 3.

Since diagnosis of osteoporosis by DXA is based on a T-score at −2.5 or below at any of the recommended sites (lumbar spine, femoral neck, or total hip), the lowest T-score was taken to dichotomously assign each result to a nonosteoporotic or osteoporotic group.

### 2.3. Statistical Analysis

Data were analyzed with the commercial statistical software Prism 5.04 for Windows and InStat3 (GraphPad, San Diego, CA). The D'Agostino and Pearson Omnibus Normality test was routinely used to assess whether data departed significantly from a Gaussian distribution. If this was the case, data are presented as median (25–75 interquartile range). Otherwise, data are expressed as mean ± standard deviation. Comparison of OST scores between women with a DXA diagnosis of osteoporosis (T-score of −2.5 and below at any site) and those without it was performed with Mann-Whitney's test. Simple linear regression was employed to assess the relationship between OST score and the lowest T-score for each patient (lumbar spine, femoral neck, or total hip). A receiver operating characteristic (ROC) curve was used to assess the area under the curve (AUC), sensitivity, and specificity. Negative and positive predictive values were calculated. The diagnostic odds ratio was calculated by Chi-square test, and results are displayed as mean (95% confidence interval = CI95). Significance level was set at 0.05.

## 3. Results and Discussion

The characteristics of the sample are shown in Table 1. Out of 4,343 patients, a total of 2,513 women were classified as nonosteoporotic while the remainder 1,830 women were classified as osteoporotic. 

Among the main risk factors detected, other than advanced age or low weight, low calcium intake (less than 1000 mg/day) was found in 70% of women, essentially corroborating the result of a previous study in the same population [22]. Fragility fractures were recalled in 16,5% of the patients, sedentarism in 15%, a family history of osteoporosis in 10%, long-term glucocorticoid therapy in 6.2%, and rheumatoid arthritis in 1.8%. Twelve percent of the patients were cigarette smokers at the time of the study, but high alcohol intake was reported by less than 1%.

In Table 2 the absolute number and the proportion of women whose T-score was at −2.5 or below at the lumbar spine, the femoral neck, the total hip, or a combination of two or all three sites are shown. Of the 1,830 women with diagnosis of osteoporosis, T-scores of −2.5 or below were found in 1,207 at the lumbar spine, in 569 at the femoral neck, and in 1063 at the total hip. These figures correspond to the total number of patients with T-scores at −2.5 or below at each site. For example, the figure of 1,207 for the lumbar spine includes 557 women with T-score at −2.5 or below at the lumbar spine only, plus 125 women with T-score at −2.5 or below at both lumbar spine and femoral neck, plus 266 women with T-score at −2.5 or below at both lumbar spine and total hip plus 259 women with T-score at −2.5 or below at lumbar spine, femoral neck and total hip.

For the whole group, OST scores ranged from –11 to +15 (−11 to 7 in osteoporotic and −7 to 15 in nonosteoporotic women). Women with a diagnosis of osteoporosis had significantly lower OST scores than those without it. Median OST scores were, respectively, 0.0 (−2 to +2) versus 2.0 (0.0 to 4.0); *P* < 0.0001. 

**Figure 1:**
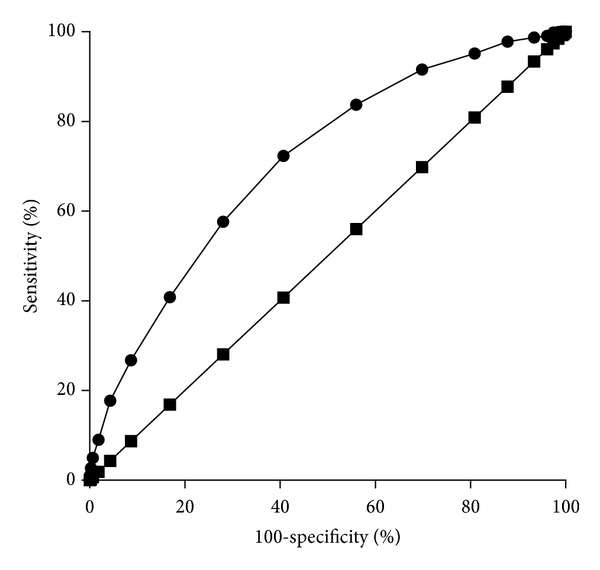
Receiver operating characteristic curve for the OST scores. The area under the curve is 0.71 (*P* < 0.0001). The straight line is the line of identity, corresponding to an area under the curve of 50%.

The result of the ROC analysis is shown in Figure 1. AUC was 0.71 (*P* < 0.0001).  Table 3 displays sensitivity and specificity for cut-off values from –3 to 3. For an OST score cut-off value of 2, the positive predictive value was 52% and the negative predictive value was 79% in the present sample. 

**Figure 2:**
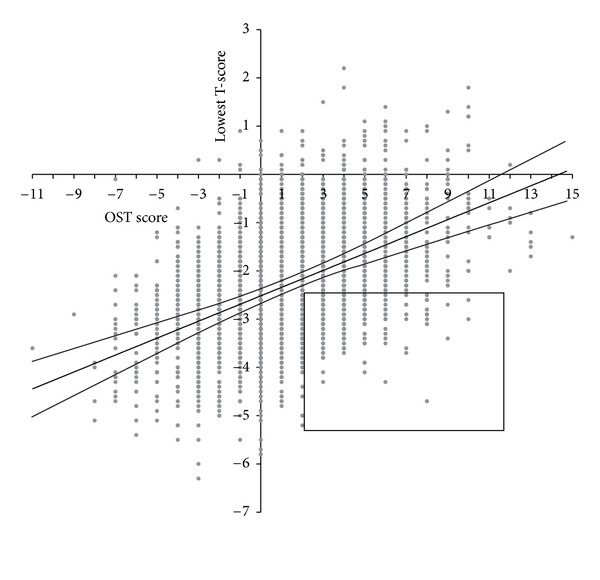
A plot of the OST score versus the lowest T-score (at any site). There is a significant linear relationship between both scores (*R* = 0.114; *P* < 0.0001). The rectangle highlights the patients with T-scores at −2.5 or below who had OST scores above 2 (*N* = 298).

Figure 2 displays OST scores versus lowest T-scores for the entire sample, showing a significant linear relationship between OST scores and lowest T-scores (*P* < 0.0001). If women with an OST score of 2 or lower are considered at high risk, and those above 2 are deemed at low risk, the unadjusted odds ratio for a diagnosis of osteoporosis by DXA of the high risk group versus the low risk group is 4.06 (CI95 3.51 to 4.71). 

The AUC obtained from a ROC analysis can range (expressed as a percentage) from 0 to 100, with 50 being the line of identity. Since sensitivity and specificity are both independent of disease prevalence, the same applies to the AUC [23]. AUC at or above 70% are deemed acceptable for a screening test. In the present study, the AUC was 71%. 

The sensitivity and specificity of any given test vary inversely according to the chosen cut-off value. In previous reports, reviewed by Rud et al. [9], the sensitivity of the OST for prediction of T-scores at −2.5 or below for any region (lumbar spine, femoral neck, or total hip) has a median of 86% (range of 53% to 95%) in white women and 82% (range 79% to 82%) in Asian women. In the present study, using a cut-off value of 2, the sensitivity in Argentinian women was 83.7%, which is intermediate between the medians for white and Asian women.

On the other hand, the specificity of the OST for any site has a median of 40% (range 34% to 72%) for white women but a higher median, of 64% (range 60% to 78%), for Asian women [9]. The estimated specificity in the present study with a cut-off at 2 was 44%, closer to the specificity for white women than for Asian women. 

The reason why values of sensitivity and particularly the specificity for Argentinian women were between those for white and Asian women is not clear, but it may be related to the fact that about 80% of the Argentine population has European ancestry, with minor but significant contributions from other ethnic groups [24]. 

One limitation of this study concerns whether the sample is representative of Mendoza's postmenopausal women. Participants referred by their physicians for BMD measurement might have more risk factors than postmenopausal women in the general population. In a recent prospective study of 720 postmenopausal women undergoing their first DXA scan, 44% were at or above 65 years of age. Of those below that age, 55% had at least one risk factor (F. D. Saraví, *unpublished data*). 

Another reason why the sample may not accurately depict the general population of postmenopausal women of Great Mendoza is socioeconomic status and educational level. Recent estimates place the fraction of the population below the poverty line at about 10% for Argentine urban areas [25]. Additionally, according to official statistics, 37% of the population does not have health insurance [26]. Although poor women or those without health insurance can still get a DXA scan through agreements between our center and the public hospital system, in practice their access is limited. These women may differ from the ones included in the present study on their educational level, nutrition, lifestyle, and prevalence of osteoporosis. 

There are several clinical instruments for the assessment of the risk of osteoporosis. Most of them consider additional factors other than age and weight, for example, the ABONE (age, bulk, No estrogen) [27]; the Osteoporosis Risk Assessment Instrument (ORAI) [5], which incorporates age range, body weight (dichotomously), and estrogen therapy; the Simple Calculated Osteoporosis Risk Estimation (SCORE), which includes race other than black, rheumatoid arthritis, nontraumatic fractures, age, weight, and estrogen therapy [28]; and the Osteoporosis Index of Risk (OSIRIS), which takes into account body weight, age, history of nontraumatic fractures, and estrogen therapy [29]. 

Comparisons of these instruments have been performed by several researchers. Geusens et al. [30] found that OST predicted bone mass equally well than ORAI and SCORE in women from the United States and the Netherlands. Similarly, a comparison performed in a large sample of Belgian women found that OST “performed as well as the more complex risk assessment indices (SCORE, ORAI, and OSIRIS) in identifying women at low risk of osteoporosis” [11]. In a 2004 review article, Wehren and Siris also stated that OST, “the simplest of the instruments, performs as well as more complex tools” [3]. Essentially the same was found in a study of 986 postmenopausal Moroccan women [31]. In a study of Canadian women, the performance of OST was as good as that of ORAI [5]. In a systematic review, it is stated that OST shows higher accuracy than ORAI and SCORE concerning the “any region” BMD target. The authors noted, however, that overall “accuracy is similar in white women, albeit the trade-off between sensitivity and specificity may differ between OST and comparator CDRs” (Clinical Decision Rules) [12]. A very recent publication compared OST, ORAI, and ABONE and reported that OST performed best in US white women [32]. In the American College of Preventive Medicine Position Statement on screening for osteoporosis it is stated about the OST, “The simplicity of this screening tool and its validation in both genders and in various races account for its popularity and widespread use in selecting patients for confirmatory BMD testing” [4].

## 4. Conclusions

In the studied sample of postmenopausal women from Mendoza, Argentina, the OST showed a performance comparable to that reported for European and US white women. The overall performance of the OST was adequate for a clinical screening method simple enough to be used both by patients and physicians. Of course, its use does not preclude careful consideration of other clinical risk factors for osteoporosis.

## Figures and Tables

**Table 1:** Characteristics of participants (*N* = 4343).

Variable	Median	Interquartile range (25–75)
Age (years)	60	54 to 67
Time since menopause (years)	12	6 to 20
Height (cm)	156.0	152.0 to 160.0
Weight (kg)	66.0	54.0 to 67.0
Body mass index (kg/m^2^)	27.1	24.3 to 30.5
OST score	1.0	−1.0 to 3.0
Lumbar spine BMD (g/cm^2^)	1.008	0.900 to 1.133
Lumbar spine T-score	−1.6	−2.5 to −0.6
Femoral neck BMD (g/cm^2^)	0.826	0.749 to 0.908
Femoral neck T-score	−1.5	−2.1 to −0.9
Total hip BMD (g/cm^2^)	0.797	0.697 to 0.904
Total hip T-score	−1.6	−2.5 to −0.8

**Table 2:** Number of women with T-score at −2.5 or below according to site.

Site(s) with a T-score ≤2.5	*N*	Percent
Lumbar spine only	557	30.4
Femoral neck only	84	4.6
Total hip only	437	23.7
Lumbar spine + femoral neck	125	6.8
Lumbar spine + total hip	266	14.5
Femoral neck + total hip	101	5.5
Lumbar spine + femoral neck + total hip	259	14.2

Total	1830	99.7

**Table 3:** Sensitivity and specificity of the OST score for predicting a T-score of −2.5 or below at any site, according to the cut-off value.

Cut-off value of the OST score	Percent sensitivity mean (CI_95_)	Percent specificity mean (CI_95_)
−3	17.7 (16.0 to 19.5)	95.7 (94.8 to 96.4)
−2	26.7 (24.7 to 28.8)	91.3 (90.2 to 92.4)
−1	40.8 (38.6 to 43.1)	83.2 (81.7 to 84.6)
0	57.6 (55.3 to 59.9)	72.0 (70.2 to 73.7)
1	72.3 (70.2 to 74.3)	59.3 (57.3 to 61.2)
2	83.7 (81.9 to 85.4)	44.0 (42.1 to 46.0)
3	91.6 (90.2 to 92.8)	30.2 (28.4 to 32.0)
